# Comparative Efficacy of Low-Carbohydrate and Ketogenic Diets on Diabetic Retinopathy and Oxidative Stress in High-Fat Diet-Induced Diabetic Rats

**DOI:** 10.3390/nu16183074

**Published:** 2024-09-12

**Authors:** Monya T. Jawharji, Ghedeir M. Alshammari, Manal Abdulaziz Binobead, Nouf Mohammed Albanyan, Laila Naif Al-Harbi, Mohammed Abdo Yahya

**Affiliations:** 1 Department of Food Science and Nutrition, College of Food and Agricultural Science, King Saud University, Riyadh 11451, Saudi Arabia; 441203713@student.ksu.edu.sa (M.T.J.); mbinobead@ksu.edu.sa (M.A.B.); lalharbi1@ksu.edu.sa (L.N.A.-H.); mabdo@ksu.edu.sa (M.A.Y.); 2 Department of Optometry, College of Applied Medical Sciences, King Saud University, Riyadh 11451, Saudi Arabia; nalbanyan@ksu.edu.sa

**Keywords:** ketogenic diet, HFD, diabetic retinopathy, rats, oxidative stress, inflammation

## Abstract

This study examined the effect of a low-carbohydrate diet (LCD) and a low-carbohydrate ketogenic diet (LCKD) on diabetic retinopathy in high-fat diet-induced diabetes mellitus in rats and studied the mechanisms of action. Rats were divided into four groups: the Control group, which was fed a normal diet for 16 weeks; the HFD group, which was fed a high-fat diet (HFD) for the first 8 weeks and then switched to a normal diet for 8 weeks; the HFD+LCD group, fed a HFD for 8 weeks followed by an LCD for 8 weeks, and the HFD+LCKD group, which was fed a HFD for 8 weeks followed by an LCKD for 8 more weeks. Both the LCD and the LCKD effectively reduced the final body and total fat weights and decreased fasting serum levels of glucose, insulin, hemoglobin A1 (HbA1C), triglycerides, cholesterol, and LDL-c. They also reduced the levels of malondialdehyde (MDA), tumor necrosis factor-α, vascular endothelial factor, caspapse-3, and bax. In the HFD rats, we found increased serum levels of β-Hydroxybutyrate and upregulated expression of Bcl2, glutathione, superoxide dismutase, and hemeoxygenase-1. Moreover, the LCD and LCKD significantly reduced mRNA levels of Kelch-like ECH-associated protein 1 (Keap1) and enhanced mRNA and nuclear concentrations of nuclear factor erythroid factor 2 (Nrf2). All these effects were associated with improved layers of the retina in the HFD − LCD and HFD + LCKD rats but not in HFD animals. The impact of the LCKD was always more profound on all measured parameters and on improving the structure of the retina compared to the LCD. In conclusion, the LCKD is superior to the LCD in preventing diabetic retinopathy in HFD-fed rats. Mechanistically, our results suggest that the hypoglycemic and hypolipidemic conditions and the Nrf2-dependent antioxidant and anti-inflammatory effects may be involved in the preventative effects of the LCD and LCKD.

## 1. Introduction

Diabetes mellitus (DM) is the most common chronic endocrine disease and is associated with a high rate of mortality due to hyperglycemia-mediated microvascular and macrovascular complications [1]. The disease can be classified into two types, type 1 DM (T1DM) and type 2 DM (T2DM), based on the etiology [1]: while T1DM is mainly attributed to the immune destruction of pancreatic beta cells, T2DM is highly linked to insulin resistance (IR), which is associated with low physical activity, an unhealthy diet, obesity, and metabolic syndrome [1,2]. However, hyperglycemia, hypertension, and low-grade inflammation remain the major factors that contribute to such diabetic complications in affected patients [2]. Diabetic retinopathy (DR) is still the most serious early complication among diabetic patients and eventually leads to visual disturbance and blindness [3]. According to the most recent reports, including 59 population-based studies, the global prevalence of DR among patients with T2DM by the end of 2020 was 22.27%, a ratio that is expected to increase in the next 10 years [4]. Major clinical findings associated with the disease include increased vascular dilation and permeability, loss of pericytes, occlusion of capillaries, proliferation, and increased thickness of the epithelium basement membranes [5,6]. Further, DR can be classified as proliferative or non-proliferative, where the latter is the phase most responsive to therapeutic efforts [5].

In searching for a suitable treatment, it becomes crucial to understand the molecular mechanisms and targets that are involved in the process of developing DR. In recent decades, accumulating evidence has shown the emerging roles of oxidative stress, inflammation, and apoptosis in the pathogenesis of DR [6,7]. The retina is one of the metabolic organs in the body that requires a constant supply of nutrients and oxygen and is also vulnerable to oxidative damage due to the inefficiency of its antioxidant defense system [7]. Currently, accumulating data have shown that the over-production of reactive oxygen species and the subsequent oxidative stress are the leading mechanisms that initiate retinal inflammation, apoptosis, and damage during the development and progression of T2DM [8,9,10,11]. Indeed, it was reported that antioxidant therapy prevented clinical abnormalities, visual loss, and retinal damage in diabetic rodents by preventing oxidative damage, neovascularization, inflammation, and apoptosis [12,13]. Systemic and retina factors usually lead to diabetic retinopathy by inducing the over-production of ROS. Hyperglycemia, inflammation, and tissue hypoxia (hypoperfusion) are the major mechanisms by which DM promotes ROS production and oxidative stress in various retinal cells [10,11,14]. Furthermore, other studies have shown that ocular factors such as the reduced antioxidant capacity of the retinal cells, which is mainly due to reduced expression and activation of the antioxidant transcription factor, nuclear factor erythroid factor-2 (Nrf2), are major independent mechanisms for the development of DR [15,16,17,18,19,20]. In support of this, Nrf2 activators are among the effective potential therapies to prevent and treat diabetic retinopathy [21].

Systemic regulation of glucose levels, drug therapy, physical exercise, diet modification, and surgical intervention remain integral parts of the management of DM and its different complications [22]. During the last two decades, several studies have shown the adverse effect of a high-carbohydrate diet (HCD) on the health and outcomes of patients with DM and have considered this type of diet a diabetogenic diet in healthy individuals, which can also lead to obesity, IR, dyslipidemia, and systemic inflammation [23,24,25]. Therefore, the shift toward a low-carbohydrate diet (LCD) has been given a higher preference as an alternative dietary intervention therapy. The low-calorie ketogenic diet (LCKD) is a common form of LCD in which the individual derives less than 13% of their total energy from carbohydrates (less than 50 g) [26]. The anti-diabetic effect of the LCD and LCKD against T2DM has been described in several studies and reviews, which have shown the great potential of this diet in reversing obesity, attenuating IR and dyslipidemia, and controlling fasting levels of glucose, HbA1c, and insulin [26,27,28,29,30,31]. In addition, ketogenesis induced by LCKD therapy exerted significant protective effects on the heart, kidney, and brain of diabetic animals and humans by modulating oxidative stress, inflammation, and apoptosis, as reported in previous studies [32,33,34,35,36,37]. It was also suggested that all the beneficial effects of the LCKD were due to its ability to mimic fasting and increase the production of ketone bodies, which can inhibit cytokine production, stimulate lipolysis, increase antioxidant synthesis, and enhance mitochondrial biogenesis by acting directly or by modulating cell signaling and the activities of certain transcription factors [34,35,36,37].

In recent years, the effectiveness of the LCKD in treating neurological disorders such as stroke, spinal cord injuries, Alzheimer’s disease, Parkinson’s disease, epilepsy, and migraines has also been reported [34,38,39]. To date, the beneficial effects of the LCKD on DR are not clear and have not been examined in depth. In a recent unique study, Chandrasekaran et al. [40] have shown that the chronic feeding of a ketogenic diet was able to reverse macular detachment in a patient with unstable proliferative diabetic retinopathy with extramacular fractional retinal detachment (TRD).

In this study, we aimed to examine and compare the protective effect of the chronic feeding of an LCD and an LCKD on the progression of DR in rats fed an HFD by targeting their effect on retinal structure, as well as on the retinal markers of oxidative stress, inflammation, and apoptosis. In addition, we aimed to understand the mechanisms of action of these diets.

## 2. Materials and Methods

All the experiments conducted in this study were approved by the Research Ethics Committee (REC) at King Saud University, Riyadh, Saudi Arabia (number KSU-SE-22-119). For the experimental procedure, adult male Wistar rats were used (7 weeks old and weighing 120 ± 20 g). They were of the same breed and were always kept in a separate room where temperature, humidity, light/dark cycle, and water supply were automatically controlled. The experimental diets were provided with no restrictions.

### 2.1. Experimental Diets

The diets included in this study were a control growth diet, a high-fat diet (HFD), an LCD, and a ketogenic diet (LCKD). All diets were formulated and purchased from Research Diets, Brunswick, NJ, USA, and their ingredients, composition, and energy equivalents are shown in Table 1.

### 2.2. Experimental Design

Four groups of rats were included in this study, each containing 16 rats. In addition, 8 rats were included per cage, thus giving 2 cages for each group of rats. The groups were designed as follows: Group 1 included rats that were fed only a normal diet for 16 weeks, and Group 2 rats were fed an HFD for the first 8 weeks and then were transferred to the control diet for the next 8 weeks. Group 3 rats were fed an HFD for the first 8 weeks and then transferred to the LCD for the next 8 weeks, and Group 4 rats were fed an HFD for the first 8 weeks and then transferred to the LCKD for the next 8 weeks. According to our preliminary data, rats fed an HFD for 8 weeks developed IR, hyperglycemia, and hyperlipidemia by the end of the first 8 weeks but required another 8 weeks with a normal diet to develop retinal damage.

### 2.3. Collection of Blood Samples

On the last day of the experiment, all animals were fasted for 10 h and then anesthetized with a ketamine/xylazine hydrochloride mix (80/10 *v*:*v*). Blood samples were collected from the right ventricle into either gel- or EDTA-containing tubes to collect serum and plasma. In all cases, blood samples were allowed to settle for 30 min at room temperature and were then centrifuged at 500× *g* for 10 min. The serum and plasma samples were aliquoted into Eppendorf tubes, labeled, and maintained at −80 °C until further biochemical analysis.

### 2.4. Collection of the Retina Samples and Fat Pads

Directly after blood collection, all rats were euthanized with cervical dislocation, and both eyes of each rat were enucleated with fine-tipped scissors to ensure minimal trauma and immediately placed in cold phosphate-buffered saline (PBS) (pH = 7.4) for preservation. Under a stereomicroscope, the cornea and lens were carefully excised using a scalpel and fine forceps to expose the retina. The retina was then carefully detached from the underlying retinal pigment epithelium (RPE) using fine-tipped forceps and a blunt dissection tool, ensuring the preservation of tissue integrity. The isolated retina was cut into smaller pieces, parts of which were frozen at −80 °C while other parts were preserved in 10% buffered formalin for histological processing. In addition, all fat pads, including the subcutaneous, peritoneal, epididymal, and mesenteric fat pads, were collected, weighed, and kept at −80 °C. The adiposity index (%) was calculated by using the following formula: (The sum of fat pads divided by final body weights) × 100.

### 2.5. Preparation of Retina Homogenates

Parts of the frozen retinas were homogenized in 4 volumes in ice-cold neutral phosphate-buffered saline to prepare total cell homogenates, which were later used for the measurement of some biochemical parameters. Other parts were used to extract the nuclear and cytoplasmic proteins by using a commercial isolation kit (number NT-032; Invent Biotechnologies, Plymouth, MN, USA).

### 2.6. Biochemical Analyses of Blood Samples

Fasting plasma glucose, insulin, and HbA1C levels, as well as the serum levels of total cholesterol (CHOL), triglycerides (TGs), high-density lipoprotein cholesterol (HDL-c), low-density lipoprotein cholesterol (LDL-c), and free fatty acids (FFAs), were measured by using ELISA kits (number 10009582 (Cayman Chemicals, Solana Beach, CA, USA); number 589501 (Ann Arbor, Dallas, TX, USA), number 80300 (Crystal Chem, Elk Grove Village, IL, USA), number EK720559 (AFG Scientific, Northbrook, IL, USA), number EK720636 (AFG Scientific, Northbrook, IL, USA), number (Ann Arbor, Dallas, TX, USA), number EK720763 (AFG Scientific, Northbrook, IL, USA), and number EK721336 (Ann Arbor, Dallas, TX, USA), respectively). The serum levels of β-hydroxybutyrate were measured with a rat-specific ELISA kit (number EK721731; AFG Scientific, Northbrook, IL, USA). Peripheral insulin resistance was calculated with the homeostasis model assessment of insulin resistance index (HOMA-IR index) by using the following equation: HOMA-IR = ([glucose] × [insulin]/405). All measurement protocols were performed for 8 samples/group as per the providers’ instructions. Control samples of known concentrations were used for normalizing the samples.

### 2.7. Biochemical Analyses of Retina Total Homogenates, Cytoplasmic Fraction, and Nuclear Fraction

The following ELISA kits were used to measure the levels of necrosis factor-α (TNF-α) and interleukin-6 (IL-6) in the retina homogenates (number ab100785 (Abcam, Cambridge, UK) and number R6000B (R&D System, Minneapolis, MN, USA), respectively). ELISA kits purchased from AFG Scientific (Northbrook, IL, USA) were used to measure the retinal homogenate levels of malondialdehyde (MDA) (number EK720188), total glutathione (GSH) (number EK720816), heme oxygenase-1 (HO-1) (number EK720658), and superoxide dismutase (SOD) (number EK720889). The total levels of the advanced glycation end-products (AGEs) were measured by using the CusaBio ELISA kit (number CSB-E09413r; St. Louis, MO, USA). Additional ELISA kits were used to measure the total levels of Bax (number E4513; BioVision, Milpitas, CA, USA), Bcl2 (number LS-F11016; LS Bio, Shirley, MA, USA), and caspase-3 (number LS-F4135; LS Bio, Shirley, MA, USA). All analyses were performed according to each manufacturer’s instructions and for *n* = 8 samples/group. The cytoplasmic and nuclear levels of Nrf2 and the nuclear levels of NF-κB in the retina extracts were determined with ELISAs (number 50296 and number 31102, respectively; Active Motif, Carlsbad, CA, USA). The total levels of Kelch-like ECH-associated protein 1 (Keap1) in the cytoplasmic extract were measured by using an ELISA kit (number MBS7218529, MyBioSource, San Diego, CA, USA). All procedures were performed for 8 samples/group as per the manufacturers’ instructions. The results were normalized using the standard curve provided and generated with each kit and presented relative to the total tissue weight of each sample.

### 2.8. Real-Time PCR in the Retina Tissues

qPCR was used to evaluate the mRNA expression of Nrf2, NF-κB, Keap1, and beta-actin (a reference gene) in the retinas of all groups of rats. The primer gene number, forward and reverse sequences, and amplification size were described in our previous study [41,42]. RNA was isolated from the frozen retina samples using a commercial RNA extraction kit (number 74104; Qiagen, Venlo, The Netherlands). First-strand cDNA was synthesized using a commercial kit (number K1621; Thermo Fisher kit, Waltham, MA, USA). All amplification reactions were conducted using the SsoFast EvaGreen Supermix kit (number 172-5200; Bio-Rad, Hercules, CA, USA) in a CFX96 real-time PCR machine (Bio-Rad, Hercules, CA, USA) as per the manufacturer’s instructions and as previously described [43]. The transcription levels of each target were presented as normalized to β-actin. All amplification reactions were performed for *n* = 6 samples/group.

### 2.9. Hematoxylin and Eosin Staining

The histological protocol was routinely performed in our laboratories [42]. The retina tissue samples were fixed for 24 h in 10% buffered formalin and were then treated with ethanol, embedded in paraffin, and cut using a microtome in sections of 3–5 μm. They were then routinely stained with H&E and were examined and photographed under a light microscope.

### 2.10. Statistical Analysis

The data of all parameters were collected and analyzed with GraphPad Prism analysis software (version 8; Solana Beach, CA, USA). All parameters were analyzed using a 2-way ANOVA test and Tukey’s post hoc test. The data were considered significantly different at *p* < 0.05 and were presented or graphed as means ± standard deviation (SD).

## 3. Results

### 3.1. Effects of Carbohydrate Calorie Restriction on Selected Diabetic Markers in HFD-Fed Rats

The final body weight, food intake, fasting plasma glucose levels, fasting insulin levels, HBA1C and HOMA-IR levels, and adiposity index, as well as the weights of the mesenteric, subcutaneous, peritoneal, and epididymal fat pads, were significantly increased in the HFD-fed rats compared to the control rats (Table 2). There were significant reductions in all these markers in the HFD + LCD- and HFD + LCKD-fed rats compared with the HFD-fed rats (Table 2). However, the reductions in final body weight, food intake, fasting plasma glucose level, fasting insulin levels, HBA1C and HOMA-IR levels, the weights of all fat pads, and the adiposity index were more significant in the HFD + LCKD-fed rats compared with the LCD-fed rats. The levels of all these markers were not significantly different when the HFD + LCDK-fed rats were compared to the control rats (Table 2). Interestingly, calorie intake was significantly higher in HFD-fed rats compared to control rats, whereas they showed no significant variations with the calorie intake measured in both the HFD + LDK- and HFD + LCDK-fed rats.

### 3.2. Effects of Carbohydrate Calorie Restriction on the Lipid Profiles and Serum Levels of β-Hydroxybutyrate in HFD-Fed Rats

The serum fasting levels of TGs, CHOL, FFAs, and LDL-c were significantly higher, but those of HDL-c were significantly lower in the HFD-fed rats than in the control rats (Table 3). There was no significant change in the levels of β-hydroxybutyrate (β-HB) between the control and HFD-fed rats (Table 3). The serum fasting levels of TGs, CHOL, FFAs, and LDL-c were significantly lower, whereas those of HDL-c and β-HB were significantly higher in both the HFD + LCD- and HFD + LCKD-fed rats. In addition, the levels of TGs, CHOL, FFAs, and LDL-c were significantly lower, and those of HDL-c and β-HB were significantly higher in the HFD + LCKD-fed rats compared with the HFD + LCD-fed rats.

### 3.3. Effects of Carbohydrate Calorie Restriction on Selected Markers of Oxidative Stress and Inflammation in Retinas of HFD-Fed Rats

**Figure 1:**
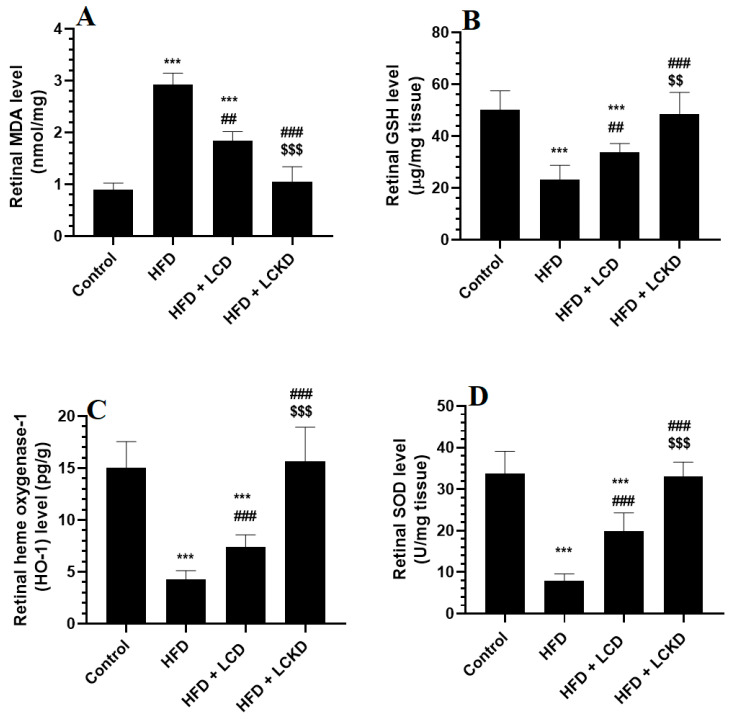
The levels of malondialdehyde (MDA) (**A**), glutathione (GSH) (**B**), heme oxygenase-1 (HO-1) (**C**), and superoxide dismutase (**D**) in retinas of all groups of rats. Data are presented as means ± SD for *n* = 8 rats/group. ***: significantly different compared with control at *p* < 0.001; ^##^ and ^###^: significantly different compared with HFD-fed rats at *p* < 0.01 and *p* < 0.001, respectively; ^$$^ and ^$$$^: significantly different compared with HFD + LCD-fed rats at *p* < 0.01 and *p* < 0.001, respectively. Malondialdehyde (MDA), total glutathione (GSH), heme oxygenase-1 (HO-1), and superoxide dismutase (SOD).

**Figure 2:**
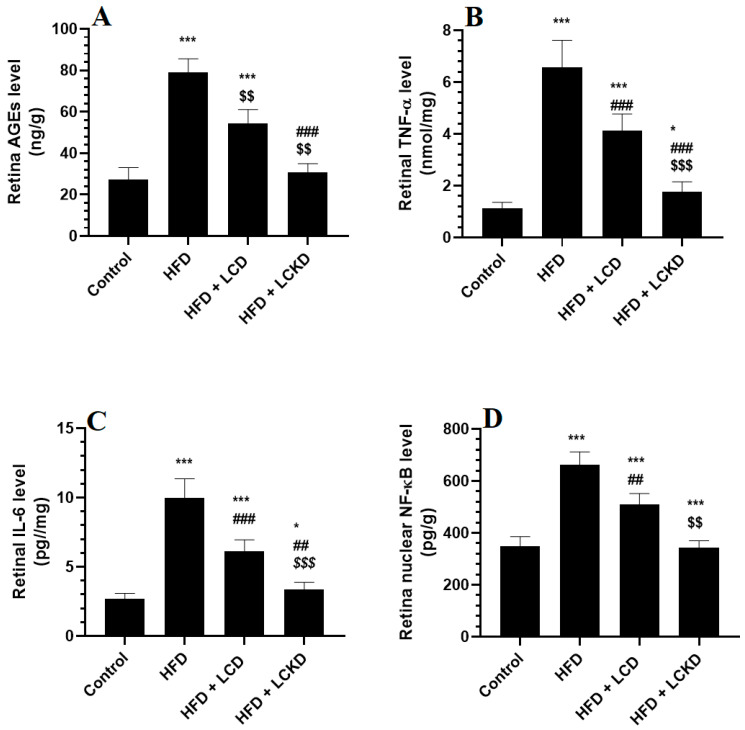
Levels of advanced aged glycation products (AGEs) (**A**), tumor necrosis factor-α (TNF-α) (**B**), levels of interleukin-6 (IL-6) (**C**), and nuclear activity of NF-κB (**D**) in retinas of all groups of rats. Data are presented as means ± SD for *n* = 8 rats/group. * and ***: significantly different compared with control at *p* < 0.05 and *p* < 0.001, respectively; ^##^ and ^###^: significantly different compared with HFD-fed rats at *p* < 0.01 and 0.001, respectively; ^$$^ and ^$$$^: significantly different compared with HFD + LCD-fed rats at *p* < 0.01 and *p* < 0.001, respectively. Tumor necrosis factor-α (TNF-α), interleukin-6 (IL-6), and nuclear factor kappa beta (NF-κB).

The levels of MDA were significantly higher and those of GSH, SOD, and HO-1 were significantly lower in the retinas of HFD-fed rats compared with control rats (Figure 1A–D). In the same manner, the retinas of HFD-fed rats showed significantly higher levels of AGEs, TNF-α, and IL-6 and significantly higher nuclear levels of NF-κB (Figure 2A–D). The alterations in all these markers of oxidative stress and inflammation were significantly reversed in the retinas of both the HFD + LCD- and HFD + LCKD-fed rats (Figure 1A–D and Figure 2A–D). However, significantly lower levels of MDA, TNF-α, and IL-6 and nuclear levels of NF-κB, as well as higher levels of GSH, SOD, and HO-1, were seen in the retinas of the HFD + LCKD-fed rats compared with the HFD + LCD-fed rats (Figure 1A–D and Figure 2A–D). Except for TNF-α and IL-6, which remained slightly higher, the levels of all remaining parameters were not significantly different between the control and HFD + LCKD-fed rats.

### 3.4. Effects of Carbohydrate Calorie Restriction on Selected Markers of Apoptosis in Retinas of HFD-Fed Rats

**Figure 3:**
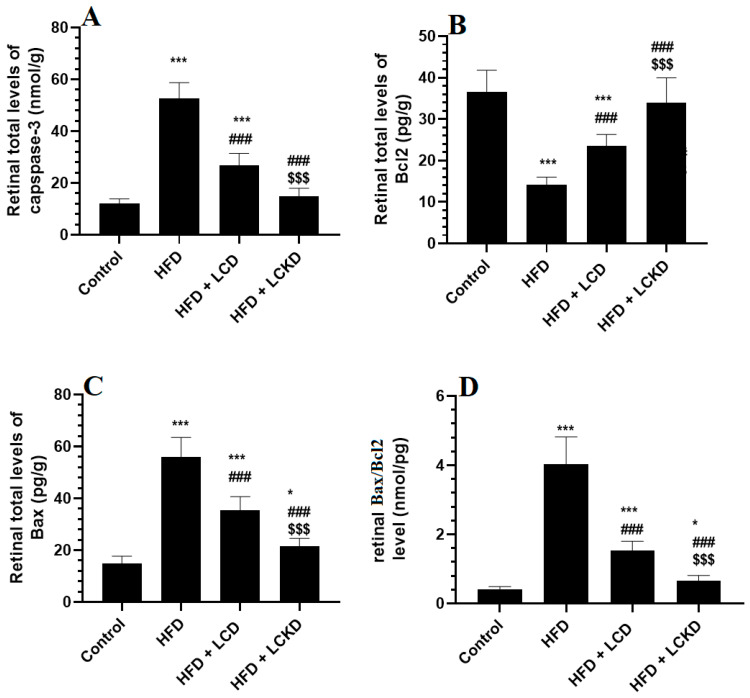
Levels of caspase-3 (**A**), Bcl2 (**B**), BAX (**C**), and Bax/Bcl2 (**D**) in the retinas of all groups of rats. Data are presented as means ± SD for *n* = 8 rats/group. * and ***: significantly different compared with control at *p* < 0.05 and *p* < 0.001, respectively; ^###^: significantly different compared with HFD-fed rats at *p* < 0.001; ^$$$^: significantly different compared with HFD + LCD-fed rats at *p* < 0.001.

The retinal levels of Bcl2 were significantly reduced, but those of caspase-3, Bax, and Bax/Bcl2 were significantly increased in the retinas of the HFD-fed rats compared with the control rats (Figure 3A–D). A significant increment in the levels of Bcl2 that was parallel with a significant decline in the levels of Bax and caspase-3, as well as in the ratio of Bax/Bcl2, was observed in the retinas of the HFD + LCD- and HFD + LCKD-fed rats compared with the HFD-fed rats (Figure 3A–D). The levels of Bcl2 were significantly higher but those of caspase-3, bax, and Bax/Bcl2 were significantly lower in the retinas of the HFD + LCKD-fed rats compared with the HFD + LCD-fed rats (Figure 3A–D).

### 3.5. Effects of Carbohydrate Calorie Restriction Markers on the Keap1/Nrf2 Axis in Retinas of HFD-Fed Rats

**Figure 4:**
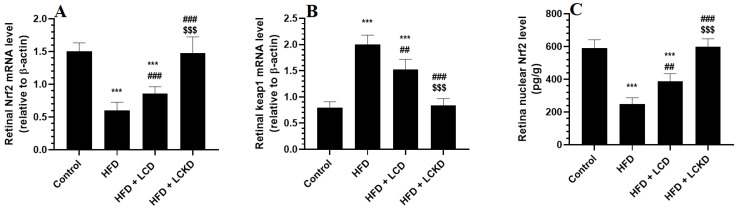
mRNA levels of Nrf2 (**A**) and Keap1 (**B**), and nuclear levels of Nrf2 (**C**) in retinas of all groups of rats. Data are presented as means ± SD for *n* = 8 rats/group. ***: significantly different compared with control at *p* < 0.001; ^##^ and ^###^: significantly different compared with HFD-fed rats at *p* < 0.01 and *p* < 0.001, respectively; ^$$$^: significantly different compared with HFD + LCD-fed rats at *p* < 0.001.

The mRNA levels of keap1 were significantly reduced, whereas those of keap1 were significantly increased in the retinas of the HFD-fed rats compared with the control rats (Figure 4A,B). In addition, the retinas of the HFD-fed rats showed a significant reduction in the nuclear retinal levels of Nrf2 compared with the control rats (Figure 3C). The mRNA levels of Keap1 were significantly decreased but the mRNA and nuclear protein levels of Nrf2 were significantly increased in the retinas of both the HFD + LCD- and HFD + LCKD-fed rats (Figure 4A–C). The reduction in the mRNA levels of Keap1 and the increments in the mRNA and nuclear protein levels of Nrf2 were more significant in the retinas of the HFD + LCKD-fed rats compared with the HFD + LCD-fed rats.

### 3.6. Effects of Carbohydrate Calorie Restriction Markers on Retina Structure

**Figure 5:**
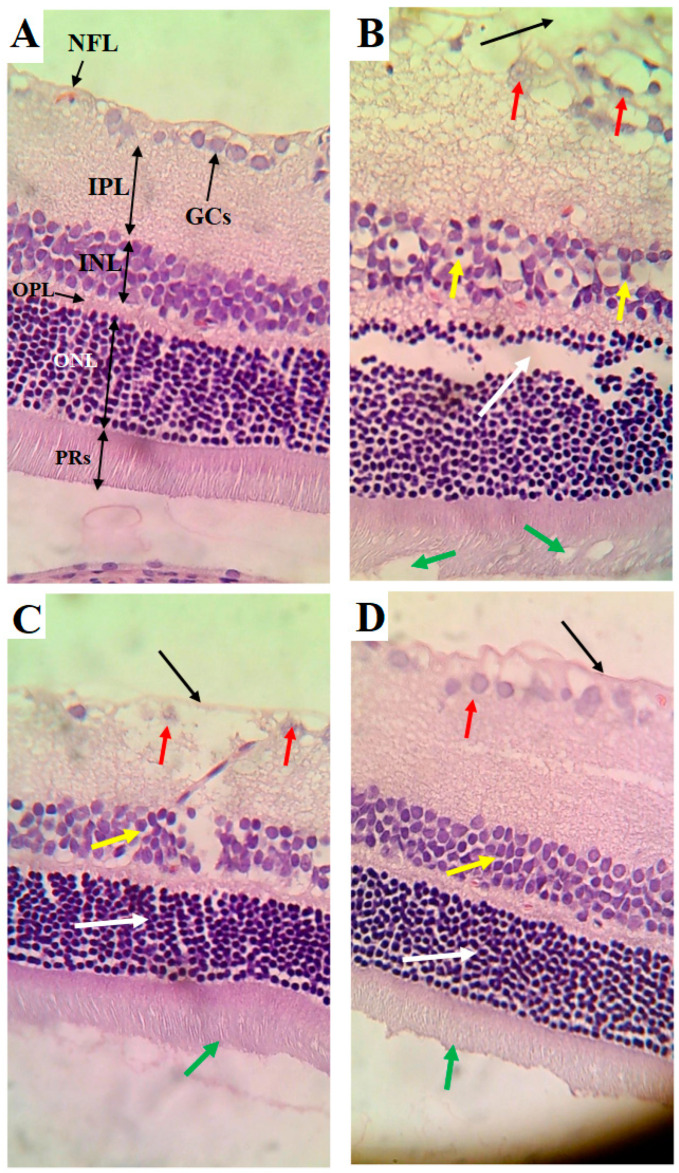
Histological alterations in retinas of all groups of rats. (**A**) Control rat: normal retina composed of intact nerve fiber layer (NFL), ganglionic cell layer (GCs), inner nuclear layer (INL), outer plexiform layer (OPL), outer nuclear layer (ONL), and photoreceptor layer (PRs). Note the abundancy of cells in each layer. (**B**) HFD-fed rat: clear increase in thickness of all layers of retina, damaged NFL (black arrow), reduced number of GCs and increased number of pyknotic GCs (red arrow), reduction in number of pyknotic cells forming the INL (yellow arrow), and vacuolization and loss of PRs (white arrow). However, cells forming the ONL appeared normal but increased in number (green arrow). (**C**) HFD + LCD rat: intact NFL (black arrow), normal ONL (white arrow), and intact PRs (green arrow). However, pyknotic GCs (red arrow) with partial loss of cells of the INL (yellow arrow) are still seen. (**D**) HFD + LCKD rat: normal retinal features with intact NLF (black arrow), abundant and intact GCs (red arrow), and intact cells forming the INL (yellow arrow), ONL (white arrow), and PRs (green arrow).

The retinas obtained from the control rats showed an intact nerve fiber layer (NFL), ganglionic cell layer (GCs), inner nuclear layer (INL), outer plexiform layer (OPL), outer nuclear layer (ONL), and photoreceptor layer (PRs) (Figure 5A). The cells forming all these layers were intact and abundant (Figure 5A). The retinas of the HFD-fed rats showed several pathological alterations, including a clear increase in the thickness of all layers forming the retina, a damaged NFL, the loss of many GCs, an increased number of pyknotic GCs, a reduction in the number of pyknotic cells forming the INL, and vacuolization and loss of PRs (Figure 5B). However, the cells forming the ONL appeared normal in the retinas of the HFD-fed rats but were increased in number (Figure 5B). The retinas of the HFD + LCD rats showed some improvement, where the NFL seemed normal and intact (Figure 5C). In addition, the ONL and PRs appeared normal (Figure 5C). However, pyknotic GCs with partial loss of the cells of the INL were still found in the retinas of this group of rats. An almost normal retinal structure was observed in the HFD + LCDK-fed rats (Figure 5D).

## 4. Discussion

Our study investigated the impact of different dietary interventions on DR in a rat model that initially underwent a high-fat diet (HFD) for 8 weeks. The rats were then switched to a normal diet for another 8 weeks. Notably, DR developed in the period following the cessation of the HFD (8 weeks later). In this context, both the low-carbohydrate diet (LCD) and the low-carbohydrate ketogenic diet (LCKD) were evaluated for their protective effects against DR. The results demonstrate that both the LCD and LCKD effectively prevented the onset of DR when administered after the HFD period, highlighting their role in the protection rather than treatment of pre-existing DR. Our findings underscore that the protective effects of the LCD and LCKD are substantial. Both diets, when introduced following the initial HFD phase, successfully mitigated the progression of DR, thus demonstrating their capability to guard against the disease rather than reverse its course. The data reveal that both diets prevented the exacerbation of body weight gain, IR, hyperglycemia, hyperinsulinemia, and dyslipidemia, which are typically associated with HFD consumption. Among these, the LCKD exhibited superior protective efficacy. The LCKD, in particular, provided significant protection against retinal damage by reducing lipid peroxidation and enhancing retinal antioxidant defenses. This was evident through increased levels of GSH, SOD, and HO-1. Furthermore, the LCKD suppressed inflammation and apoptosis markers, which were linked to the repression of NF-κB, a key transcription factor involved in inflammatory responses. This diet also stimulated Nrf2 transcription and its nuclear activation more effectively than the LCD by inhibiting Keap1. Elevated β-hydroxybutyrate (β-HB) levels associated with the LCKD contributed significantly to its protective effects, given β-HB’s known roles in reducing hyperglycemia, oxidative stress, and inflammation.

The HFD model effectively induces obesity, metabolic syndrome, and T2DM, conditions that are significant risk factors for DR and replicate features seen in humans [43]. During the HFD phase, the rats experienced various metabolic disturbances, including sustained hyperglycemia, IR, and increased inflammatory cytokines from adipose tissue [14]. Hyperglycemia, in particular, induces oxidative stress through ROS, which exacerbates retinal damage by promoting angiogenesis, inflammation, and apoptosis [44]. Similarly, hyperlipidemia, characterized by elevated levels of FFA, cholesterol, and triglycerides, contributes to DR by further increasing oxidative stress and damaging retinal cells [45,46]. These observations are consistent with the documented relationship between high lipid levels and DR severity [46,47,48,49,50]. Associated with the obvious retinal damage, all these metabolic abnormalities were also observed in the HFD-fed rats in this study, thus validating our animal model. However, the retinal protective effects observed with both LCD and LCKD diets were attributed to their ability to reduce body weight gain and improve metabolic parameters, thus alleviating oxidative stress in the retina. The LCKD’s superior protective effect aligns with previous studies demonstrating its greater efficacy compared to the LCD in mitigating obesity and improving metabolic outcomes [51,52,53,54,55,56]. Additionally, reports indicate that the LCKD’s protective benefits on lifespan and mortality rates surpass those of the LCD [57].

The mechanisms underlying the protective effects of the LCKD include the production of β-HB, which reduces appetite and food intake by stimulating anorexigenic substances such as leptin and peptide YY while inhibiting orexigenic substances such as ghrelin [58,59,60,61,62]. β-HB also enhances energy expenditure and reduces fat accumulation by stimulating lipolysis and suppressing adipogenesis [63]. These effects are consistent with observations that the LCKD promotes more rapid weight loss compared to the LCD, especially due to its impact on diuresis and glycogenolysis during the early phases of the diet [64,65,66].

Oxidative stress, driven by hyperglycemia-derived ROS, plays a critical role in DR pathogenesis [10,11,45,67]. ROS, generated through glucose autoxidation, mitochondrial damage, and inflammatory pathways, contribute to retinal damage by promoting autophagy, pyroptosis, inflammation, and apoptosis [65,66]. The data from our study align with prior findings showing that antioxidant therapies can mitigate DR progression and protect against retinal damage [44,45,46,49]. Both LCD and LCKD diets improved retinal antioxidant capacity, reduced AGE formation, and alleviated oxidative stress and inflammation, thereby supporting the effectiveness of carbohydrate restriction in managing oxidative stress [68,69,70,71]. Both diets facilitated antioxidant defense mechanisms via Nrf2 activation, which enhances the expression of antioxidant genes such as HO-1, catalase, and SOD while inhibiting NF-κB [64,72]. However, the LCKD was more effective in stimulating Nrf2 signaling and improving antioxidant levels than the LCD. The enhanced impact of the LCKD is likely due to higher β-HB levels, which further activate Nrf2 and reduce oxidative stress [73,74]. Previous research supports Nrf2 activation as a key mechanism in mitigating oxidative stress and protecting against various conditions [75].

The LCKD’s ability to enhance antioxidant defenses and reduce oxidative damage is also linked to its role in calorie restriction. Carbohydrate reduction through calorie restriction has been shown to activate the Nrf2/antioxidant axis, thereby alleviating oxidative stress [75]. The higher expression of Nrf2 and antioxidant genes observed in the retinas of rats fed an LCKD is consistent with studies showing improved Nrf2 activity and mitochondrial efficiency associated with ketogenic diets [75,76]. β-HB’s role in scavenging free radicals, stimulating mitochondrial uncoupling and biogenesis, and suppressing mitochondrial permeability transition supports its protective role against oxidative stress and inflammation [77,78,79].

## 5. Conclusions

In conclusion, our study robustly demonstrates that both LCD and LCKD diets offer significant protective effects against DR in rats previously fed an HFD, with the LCKD showing greater efficacy. These diets, administered after the HFD phase, successfully prevented the development of DR, underscoring their role as preventive rather than therapeutic interventions. The protection provided by these diets is mediated through the reversal of hyperglycemia, obesity, and IR, as well as the suppression of oxidative stress and inflammation via Nrf2/antioxidant axis activation and NF-κB inhibition. The superior protective effect of the LCKD highlights its potential as a more effective dietary intervention for preventing diabetic complications.

## 6. Study Limitations and Future Directions

Our study presents valuable insights but has several limitations. The study’s 16-week duration, with 8 weeks on an HFD followed by 8 weeks on an LCD or LCKD, might not capture the long-term effects of these diets on DR. Extended study periods are needed to evaluate the sustainability and long-term outcomes of these dietary interventions. Although the HFD-induced rat model is useful, it may not fully replicate the complexity of human DR, necessitating validation through human trials or more advanced animal models to enhance applicability. Our investigation was limited to specific biomarkers, which may not provide a comprehensive picture of DR pathology; future research should incorporate detailed histopathological and functional assessments of retinal health. Additionally, variability in LCD and LCKD formulations across studies highlights the need for standardized dietary protocols to ensure consistency in results. Investigating the molecular mechanisms underlying the observed effects, particularly the roles of the Nrf2 and NF-κB pathways, is essential for understanding the full scope of these diets’ protective mechanisms. Future studies should also consider the impact of these diets on other metabolic parameters and their potential side effects. Long-term and comparative studies will be crucial for assessing the overall efficacy and safety of the LCD and LCKD in DR management. In addition, translating these findings into clinical practice will require rigorous human trials and consideration of individualized dietary approaches to optimize DR prevention and treatment strategies. Moreover, a critical aspect of our research was ensuring that caloric intake variations did not confound the observed effects of the dietary interventions. We recognize that differences in caloric intake among groups can influence metabolic outcomes and potentially skew results. To address this concern, we monitored and standardized caloric intake across all experimental groups. By providing adjusted food quantities and regularly measuring intake, we aimed to keep the total caloric intake as consistent as possible among the different diet groups. This step was crucial in isolating the effects of dietary composition from those of caloric consumption. However, we admit that one notable limitation of our study is the absence of a caloric restriction control group. Although we controlled caloric intake to the best of our ability, we did not include a separate group subjected solely to caloric restriction without dietary composition changes. As a result, it is possible that some of the observed effects may be partially attributed to caloric restriction rather than the specific effects of an LCD or LCKD. Future studies should consider including a caloric restriction group to evaluate the impact of reduced caloric intake separately and better distinguish its effects from those of diet composition. Finally, normalizing data by tissue weight assumes uniform tissue morphology across groups, which may not fully account for the significant morphological differences observed in this study. This limitation could impact the interpretation of results, and future studies should consider alternative normalization methods, such as DNA content or housekeeping proteins, to address these variations.

## Figures and Tables

**Table 1:** Composition and components of the animals’ diets used in the experiment.

Ingredients #	LCKD		LCD		HFD		Control Diet	
	g%	kcal%	g%	kcal%	g%	kcal%	g%	kcal%
Protein	31	20	26	20	24	20	19	20
Carbohydrate	0	0	26	20	41	35	67	70
Fat	54	80	35	60	24	45	4	10
Total		100		100		100		100
kcal/g	6.1		5.24		4.73		3.8	
Ingredient	g	kcal	g	kcal	g	kcal	g	kcal
Proteins								
Casein	200	800	200	800	200	800	200	800
L-Cystine	3	12	3	12	3	12	3	12
Carbohydrates								
Corn Starch	0	0	0	0	72.8	291	550	2200
Maltodextrin 10	0	0	125	500	100	400	150	600
Sucrose	0	0	68.8	500	172.8	291	0	0
Fibers								
Cellulose	50	0	50	50	50	0	50	0
Fats								
Soybean Oil	25	225	25	225	25	225	25	225
Lard	335	3015	245	2205	177.5	177.5	20	180
Others								
Mineral Mix, S10026	10	0	10	0	10	0	10	0
DiCalcium Phosphate	13	0	13	0	13	0	13	0
Calcium Carbonate	5.5	0	5.5	0	5.5	0	5.5	0
Potassium Citrate, 1 H_2_O	16.5	0	16.5	0	16.5	0	16.5	0
Vitamin Mix, V10001	0	0	10	40	10	40	10	40
Vitamin Mix V10001C	1	4	0	0	0	0	0	0
Choline Bitartrate	2	0	2	0	2	0	2	0
FD&C Red Dye #40	0	0	0.05	0	0.05	0	0.025	0
FD&C Blue Dye #1	0	0	0	0	0	0	0.025	0
Total	661	4056	773.85	4057	858.15	4057	1055.05	4057

**Table 2:** Changes in diabetic parameters among all groups of rats.

Parameter	Control	HFD + Normal Diet	HFD + LCD	HFD + LCKD
Final body weight (g)	482.2 ± 39.2	612.3 ± 55.4 ***	512.2 ± 44.9 *^,##^	473.4 ± 44.3 ^##,$$$^
Food intake (g/rat/day)	32.6 ± 3.1	49.4 ± 5.2 ***	40.23 ± 4.1 *^,##^	33.1 ± 2.7 ^###,$$$^
Food intake (g/rat/week)	235.2 ± 24.3	356.4 ± 73***	287.3 ± 22.1*^,##^	231.9 ± 225 ^###,$$$^
Calorie intake (Kcal/week)	874 ± 67	1665 ± 145 ***	1512.4 ± 144 ***	1420.4 ± 153 ***
Fasting plasma glucose (mg/dL)	114.3 ± 10.5	192.3 ± 18.3 ***	153.3 ± 14.0 **^,##^	117.6 ± 11.4 ^###,$$$^
Fasting insulin levels (ng/mL)	3.75 ± 0.46	6.53 ± 0.73 **	5.12 ± 0.58 **^,##^	4.11 ± 0.39 ^###,$$$^
HbA1C (%)	3.31 ± 0.31	7.61 ± 0.89 **	6.13 ± 0.71 ***^,##^	3.73 ± 0.54 ^###,$$$^
HOMA-IR	1.03 ± 0.13	3.1 ± 0.46 ***	1.93 ± 0.25 ***^,###^	1.22 ± 0.17 ^###,$$$^
Mesenteric fat (g)	5.78 ± 0.73	11.5 ± 1.7 ***	7.65 ± 0.84 **^,###^	5.92 ± 0.68 ^###,$$$^
Subcutaneous fat (g)	5.11 ± 0.65	9.78 ± 1.3 ***	6.89 ± 0.72 *^,###^	5.5 ± 0.53 ^###,$$$^
Peritoneal fat (g)	4.83 ± 0.57	10.33 ± 1.43 ***	8.44 ± 0.83 ***^,###^	5.22 ± 0.59 ^###,$$$^
Epididymal fat (g)	7.33 ± 0.63	13.2 ± 2.43 ***	9.32 ± 1.12 *^,###^	7.82 ± 0.93 ^###,$$$^
Total fat weight (g)	22.8 ± 1.7	44.5 ± 4.1 ***	31.4 ± 3.7 **^,###^	24.5 ± 2.6 ^###,$$$^
Adiposity index (%)	4.71 ± 0.37	7.6 ± 0.78 ***	6.09 ± 0.58 **^,###^	4.99 ± 0.37 ^###,$$$^

Data are presented as means ± SD for *n* = 8 rats/group. *, **, and ***: significantly different compared to the control at *p* < 0.05, *p* < 0.01, and *p* < 0.001, respectively; ^##^ and ^###^: significantly different compared with HFD-fed rats at *p* < 0.01 and *p* < 0.001, respectively; ^$$$^: significantly different compared with HFD + LCD-fed rats at *p* < 0.001.

**Table 3:** Lipid profiles and levels of Β-hydroxybutyrate (β-HB) in the serum of all groups of rats.

Parameter	Control	HFD + Normal Diet	HFD + LCD	HFD + LCKD
TGs (mg/dL)	77.6 ± 7.8	186.7 ± 17.3 ***	108.4 ± 11.3 ***^,###^	84.3 ± 9.1 **^,###,$$$^
CHOL (mg/dL)	94.3 ± 8.7	234.3 ± 20.5 ***	143.4 ± 12.4 ***^,###^	101.3 ± 11.5 ^###,$$$^
LDL-c (mg/dL)	41.2 ± 4.9	110.2 ± 9.8 ***	75.4 ± 8.3 ***^,###^	52.2 ± 6.4 *^,###,$$$^
HDL-c (mg/dL)	32.2 ± 2.9	15.7 ± 1.9 ***	22.6 ± 1.8 ***^,###^	34.3 ± 3.1 ^###,$$$^
FFAs (µmol/L)	324.3 ± 43.2	654.3 ± 47.8 ***	492.2 ± 37.8 **^,###^	365.3 ± 47.6 ^###,$$$^
β-Hydroxybutyrate (μM/L)	56.5 ± 6.4	51.3 ± 5.8 ***	85.7 ± 7.1 *^,###^	134.2 ± 11.3 ^###,$$$^

Data are presented as means ± SD for *n* = 8 rats/group. *, **, and ***: significantly different compared to the control at *p* < 0.05, *p* < 0.01, and *p* < 0.001, respectively; ^###^: significantly different compared with HFD-fed rats at *p* < 0.001; ^$$$^: significantly different compared with HFD + LCD-fed rats at *p* < 0.001.

## Data Availability

The datasets used and analyzed in the current study are available from the corresponding author upon reasonable request.
